# Use of, time to, and type of first add‐on anti‐hyperglycaemic therapy to metformin in Australia, 2018–2022

**DOI:** 10.1111/bcp.16231

**Published:** 2024-09-03

**Authors:** Tamara Y. Milder, Jialing Lin, Sallie‐Anne Pearson, Jerry R. Greenfield, Richard O. Day, Sophie L. Stocker, Brendon L. Neuen, Michael O. Falster, Juliana de Oliveira Costa

**Affiliations:** ^1^ Medicines Intelligence Research Program, School of Population Health, Faculty of Medicine and Health University of New South Wales Sydney Australia; ^2^ Department of Diabetes and Endocrinology, St. Vincent's Hospital Sydney Australia; ^3^ Department of Clinical Pharmacology and Toxicology, St. Vincent's Hospital Sydney Australia; ^4^ Clinical Diabetes, Appetite and Metabolism Laboratory Garvan Institute of Medical Research Sydney Australia; ^5^ School of Clinical Medicine, UNSW Medicine & Health, St Vincent's Healthcare Clinical Campus University of New South Wales Sydney Australia; ^6^ School of Pharmacy, Faculty of Medicine and Health University of Sydney Australia; ^7^ The George Institute for Global Health University of New South Wales Sydney Australia; ^8^ Department of Renal Medicine, Royal North Shore Hospital Sydney Australia

**Keywords:** combination therapy, GLP‐1 receptor agonists, pharmacoepidemiology, pharmacotherapy, SGLT2 inhibitors, type 2 diabetes

## Abstract

**Aims:**

The aim of this study was to examine contemporary trends in the use of, time to, and type of first add‐on anti‐hyperglycaemic therapy to metformin in Australia.

**Methods:**

We used the dispensing records of a 10% random sample of Pharmaceutical Benefits Scheme (PBS) eligible people. We included people aged 40 years and older initiating metformin from 1 January 2018 to 31 December 2020. Our primary outcome was first add‐on anti‐hyperglycaemic medicine within 2 years of metformin initiation. We analysed time to dispensing of first add‐on therapy. All analyses were stratified by metformin initiation year.

**Results:**

Overall, 38 747 people aged 40 years and older initiated metformin between 2018 and 2020. Approximately one‐third (*n* = 12 946) of people received add‐on therapy with the proportion increasing slightly by year of metformin initiation (32.3% in 2018 to 34.8% in 2020). Amongst people with add‐on therapy following metformin initiation, sodium‐glucose cotransporter 2 inhibitor (SGLT2i) use increased from 28.8% (2018) to 35.0% (2020), and glucagon‐like peptide‐1 receptor agonists (GLP‐1 RA) increased from 3.0% to 9.6%, respectively. Dipeptidyl peptidase‐4 inhibitors and sulfonylureas as first add‐on therapy decreased and insulin remained stable. One‐third of people with add‐on therapy initiated the therapy on the same day metformin was initiated, i.e. initial combination therapy.

**Conclusions:**

Amongst people initiating metformin from 2018 to 2020, there was an increasing proportion of SGLT2i and GLP‐1 RA being used as first add‐on therapy. However, the overall prevalence of add‐on therapy was low. Advocacy to promote add‐on therapy with cardiorenal beneficial medicines is critical to reduce type 2 diabetes morbidity and mortality.

**What is already known about this subject**

- Type 2 diabetes management guidelines have changed in recent years from a focus on glycaemic control to cardiorenal risk reduction with the advent of sodium‐glucose cotransporter 2 inhibitors (SGLT2i) and glucagon‐like peptide‐1 receptor agonists (GLP‐1 RA).
- Few studies have explored first add‐on anti‐hyperglycaemic therapy to metformin.

**What this study adds**

- Only one‐third of people received add‐on therapy to metformin within 2 years of metformin initiation, commonly as initial combination therapy.
- SGLT2i and GLP‐1 RA were increasingly used as first add‐on therapy.
- Policy and prescriber level advocacy is required to promote increased use of add‐on therapy with SGLT2i and GLP‐1 RA.

## 1 INTRODUCTION

Sodium‐glucose cotransporter 2 inhibitors (SGLT2i) and glucagon‐like peptide‐1 receptor agonists (GLP‐1 RA) are two classes of type 2 diabetes medicines with significant cardiovascular, metabolic and renal benefits.^1^, ^2^ These extra‐glycaemic benefits are key because cardiovascular disease (CVD), heart failure and chronic kidney disease (CKD) are major causes of mortality and morbidity for people living with type 2 diabetes.^3^, ^4^, ^5^ As evidence of these extra‐glycaemic benefits have emerged, there have been rapid changes to international and national type 2 diabetes management guidelines with an increasing focus on cardiorenal risk reduction.^6^ In 2018 the American Diabetes Association recommended for the first time to add after metformin a medicine proven to reduce major adverse cardiovascular events and/or cardiovascular mortality in people with atherosclerotic CVD.^7^ In mid‐2020, the Australian Diabetes Society updated its Type 2 Diabetes Management Algorithm and highlighted the cardiac and renal benefits of SGLT2i, as well as the cardiovascular benefits of GLP‐1 RA.^8^ Two years later, the algorithm was updated with the recommendation for SGLT2i to be second‐line therapy after metformin in people with CVD, multiple cardiovascular risk factors and/or CKD, or GLP‐1 RA where SGLT2i were not tolerated or contraindicated.^9^

Early use of SGLT2i and GLP‐1 RA in the treatment of type 2 diabetes is critical as the cardiovascular and renal benefits are independent of the glucose‐lowering effects of these medicines, and to maximize the lifetime benefit from use of these medicines.^1^, ^10^, ^11^, ^12^ While multiple studies have identified changes in prevalent use of these medicines over time,^13^, ^14^ there have been limited studies about incident first add‐on therapy to metformin. A recent such study found sharp changes in uptake of both SGLT2i and GLP‐1 RA as first add‐on therapy in the United States and the United Kingdom, but with differences between countries in the medicine where this increase was most pronounced (SGLT2i in the United Kingdom; GLP‐1 RA in the United States).^15^ While this pattern may relate to the regulatory and financial context of prescribing, there are few international studies to compare these results. Australia has a national Pharmaceutical Benefits Scheme (PBS) providing subsidization of medicines to all Australian citizens and permanent residents. SGLT2i as first add‐on anti‐hyperglycaemic therapy to metformin has been PBS‐subsidized for many years for people with glycated haemoglobin (HbA1c) measurements >7% (53 mmol/mol). Exploring the use of these medicines in different countries with varying restriction and reimbursement policies is of clinical interest and relevance. Additionally, despite the importance of early use of diabetes medicines with cardiorenal benefits, time to first add‐on therapy has not been extensively studied.

Hence, given the considerable changes to type 2 diabetes management guidelines since 2018, this nationwide incident drug utilization study aimed to examine contemporary trends in the use of, time to, and type of first add‐on anti‐hyperglycaemic therapy in people initiating metformin. We also aimed to explore differences in first add‐on therapy across patient and prescriber characteristics.

## 2 METHODS

### 2.1 Data source

The PBS (paid for by the Australian Government) enables subsidized access to prescribed medicines for all Australian citizens and permanent residents in the community, private hospitals and on discharge from public hospitals in most states (state governments cover the cost of prescribed medicines to inpatients). We used the PBS dispensing claims for a 10% random sample of all PBS‐eligible people between 1 January 2017 and 31 December 2022.^16^ Private dispensing for medicines not listed on the PBS or not meeting PBS indications are not captured in this collection.

During the study period, the general co‐payment (amount a person pays towards the cost of a PBS‐subsidized medicine) was approximately A$40 per prescription. Many people on low incomes are eligible for a reduced (concessional) co‐payment of about A$7 per prescription, for example all social security recipients.

### 2.2 Medicines of interest

We were interested in anti‐hyperglycaemic medicine classes. We focused on the following anti‐hyperglycaemic medicine classes: metformin, SGLT2i, dipeptidyl peptidase‐4 inhibitors (DPP‐4i), sulfonylureas, GLP‐1 RA and insulin, which represent the vast majority of anti‐hyperglycaemic medicines used in Australia.^13^ To identify these medicines, we mapped PBS item codes to the World Health Organization (WHO) Anatomic Therapeutic Chemical (ATC) classification for “drugs used in diabetes” (see Table S1). Metformin, SGLT2i and DPP‐4i are available as single medicine tablets, as well as fixed‐dose combination tablets with each other. We split combination therapies into their active ingredients for analyses.

Clinicians are required to include a PBS‐specific code on a prescription to reflect indication of therapy for a patient to receive PBS‐subsidized SGLT2i, DPP‐4i or GLP‐1 RA.^16^ During the study period, SGLT2i and DPP‐4i were subsidized on the PBS for the treatment of type 2 diabetes if a patient had a HbA1c measurement of >7% (53 mmol/mol) despite treatment with metformin.^17^ GLP‐1 RA were subsidized on the PBS for the treatment of type 2 diabetes as add‐on therapy to metformin if a patient had a HbA1c > 7% (53 mmol/mol) and had a contraindication to or was intolerant of a sulfonylurea.

### 2.3 Study population and design

Our study population was a new user cohort: all adults aged 40 years and over initiating metformin between 1 January 2018 and 31 December 2020 (see Figure S1 for study design). We did not include adults aged less than 40 years because of the use of metformin to treat gestational diabetes and polycystic ovarian syndrome. We defined initiation as a new dispensing of metformin alone or in combination without a prior dispensing of metformin or another anti‐hyperglycaemic medicine in the past 365 days. We defined the index date as the first dispensing date of metformin. Individuals were included only once: the first time they met the eligibility criteria.

We followed up people from the index date to the first add‐on anti‐hyperglycaemic medicine, date of death, or 730 days, whichever occurred earliest. As the PBS data only contains a year of death, offset by ±180 days to protect patient privacy,^16^ we used a proxy to estimate the date of death during the follow‐up period.^18^ For people with a death recorded, we estimated the date as the last dispensing of any medicine within the potential range of the date of death.

### 2.4 Outcome measures

The main outcome measure was first add‐on anti‐hyperglycaemic medicine, defined as at least one dispensing while on metformin treatment (see Figure S2). As the PBS data do not include information on daily dose, we estimated the medicine exposure for each dispensing of metformin using an estimated period of exposure.^19^, ^20^ The PBS subsidizes different metformin package formulations, and we measured the intervals between dispensing for the same metformin formulations for individuals with more than one metformin dispensing. The treatment exposure was calculated as the number of days in which 75% of people had received a subsequent dispensing of metformin. The treatment exposure included the day metformin was initiated.

To define time to add‐on therapy, we calculated the time interval between the initiation of metformin and first dispensed add‐on therapy. This included initial combination therapy where metformin and add‐on anti‐hyperglycaemic medicine(s) were initiated on the same day. There may have been more than one anti‐hyperglycaemic medicine classified as first add‐on if two or more medicines were dispensed on the same day within the treatment exposure of metformin (see Person E in Figure S2).

### 2.5 Patient and prescriber characteristics

We examined individual characteristics at the index date including age, sex and PBS concessional status (people in Australia, for example pensioners, are entitled to a concessional benefit), as well as prescriber group for initiation of metformin and add‐on therapy (primary care, secondary care [for example, medical or emergency physician, psychiatrist, surgeon]). We identified selected patient morbidities using the Rx‐Risk Comorbidity Index in a 12‐month look back period (i.e. 365 days before and including the index dispensing).^21^ Selected morbidities included hypertension, hyperlipidaemia, congestive heart failure, arrhythmia, gout, renal disease, and anxiety and/or depression, use of antiplatelets and use of anticoagulants.

### 2.6 Statistical analysis

All analyses were stratified by year of metformin initiation. We described the study population according to the variables of interest. To examine use of add‐on therapy we used Kaplan–Meier methods, censoring on death. We compared the proportion of people with add‐on therapy across years of metformin initiation using the log‐rank test. We used the Kaplan–Meier plots to estimate time to add‐on therapy.

We described the characteristics of people at the time of metformin initiation who did and did not receive add‐on anti‐hyperglycaemic therapy. Of people with add‐on therapy (denominator), we calculated the proportion receiving each medicine class (SGLT2i, DPP‐4i, sulfonylureas, GLP‐1 RA, insulin; the numerator) as their first add‐on anti‐hyperglycaemic medicine to metformin. We examined type of first add‐on medicine class by: age categories (40–49, 50–59, 60–69, 70–79 and 80 + years), sex and specific comorbidities (using the Rx‐Risk Comorbidity Index). We also explored the type of prescriber group of metformin initiation, as well as add‐on therapy, amongst people who received it.

### 2.7 Sensitivity analysis

We estimated the period of exposure for each metformin dispensing to identify when another anti‐hyperglycaemic medicine was used as add‐on therapy. We tested the impact of this estimation by changing the definition of the treatment exposure of metformin to the number of days in which 90% (rather than 75%) of people received a subsequent dispensing of metformin.

All data analyses were completed using SAS version 9.4 (SAS Institute Inc., Cary, NC, USA). We used R version 4.3.1 (R Core Team 2017, Vienna, Austria) for producing figures.

This study was approved by the New South Wales Population and Health Services Research Ethics Committee (approval number 2019/ETH01776) with a waiver of individual consent. Data access was granted by the Services Australia External Request Evaluation Committee (approval number RMS2613).

### 2.8 Nomenclature of targets and ligands

Key protein targets and ligands in this article are hyperlinked to corresponding entries in http://www.guidetopharmacology.org, and are permanently archived in the Concise Guide to PHARMACOLOGY 2023/2024.^22^, ^23^

## 3 RESULTS

Our study population consisted of 38 747 people initiating metformin between 2018 and 2020 (Table 1). The median age was 60 years and over half were male. Approximately half and one‐third of the study population were dispensed lipid‐ and blood pressure‐lowering medicines, respectively, in the year prior to metformin initiation. Characteristics of the population were similar across year of metformin initiation (2018, 2019 and 2020).

**TABLE 1:** Characteristics of study population overall and by year of metformin initiation.

Characteristics	All	2018	2019	2020
	*N* = 38 747	*N* = 12 827	*N* = 12 613	*N* = 13 307
Age, years, median (IQR)	60 (51–69)	60 (51–69)	60 (51–69)	60 (51–70)
Age categories, years, *n* (% of *N*)				
40–49	8385 (21.6)	2826 (22.0)	2782 (22.1)	2777 (20.9)
50–59	10 716 (27.7)	3580 (27.9)	3458 (27.4)	3678 (27.6)
60–69	10 593 (27.3)	3483 (27.2)	3506 (27.8)	3604 (27.1)
70–79	6564 (16.9)	2128 (16.6)	2081 (16.5)	2355 (17.7)
80+	2489 (6.4)	810 (6.3)	786 (6.2)	893 (6.7)
Sex, *n* (% of *N*)				
Male	20 640 (53.3)	6785 (52.9)	6745 (53.5)	7110 (53.4)
Female	18 107 (46.7)	6042 (47.1)	5868 (46.5)	6197 (46.6)
Concessional beneficiaries, *n* (%)	18 448 (47.6)	6099 (47.6)	5942 (47.1)	6407 (48.2)
History of selected morbidities and medicine use^a^, *n* (% of *N*):				
Anticoagulants	3010 (7.8)	951 (7.4)	939 (7.4)	1120 (8.4)
Antiplatelets	2323 (6.0)	787 (6.1)	729 (5.8)	807 (6.1)
Arrhythmia	1198 (3.1)	398 (3.1)	350 (2.8)	450 (3.4)
Congestive heart failure	2248 (5.8)	672 (5.2)	742 (5.9)	834 (6.3)
Hypertension	12 591 (32.5)	4088 (31.9)	4134 (32.8)	4369 (32.8)
Hyperlipidaemia	18 864 (48.7)	6164 (48.1)	6107 (48.4)	6593 (49.6)
Gout	2658 (6.9)	843 (6.6)	875 (6.9)	940 (7.1)
Renal disease	77 (0.2)	28 (0.2)	32 (0.3)	17 (0.1)
Anxiety and/or depression	1567 (4.0)	518 (4.0)	540 (4.3)	509 (3.8)
Specialty of metformin prescriber, *n* (% of *N*)				
Primary care	22 849 (59.0)	7819 (61.0)	7753 (61.5)	7277 (54.7)
Secondary care	2002 (5.2)	671 (5.2)	741 (5.9)	590 (4.4)
Unknown/undisclosed	13 896 (35.9)	4337 (33.8)	4119 (32.7)	5440 (40.9)

Abbreviation: IQR, interquartile range.

^a^ Estimated using the Rx‐Risk Comorbidity Index.^21^

### 3.1 Add‐on therapy within 2 years of metformin initiation

**FIGURE 1:**
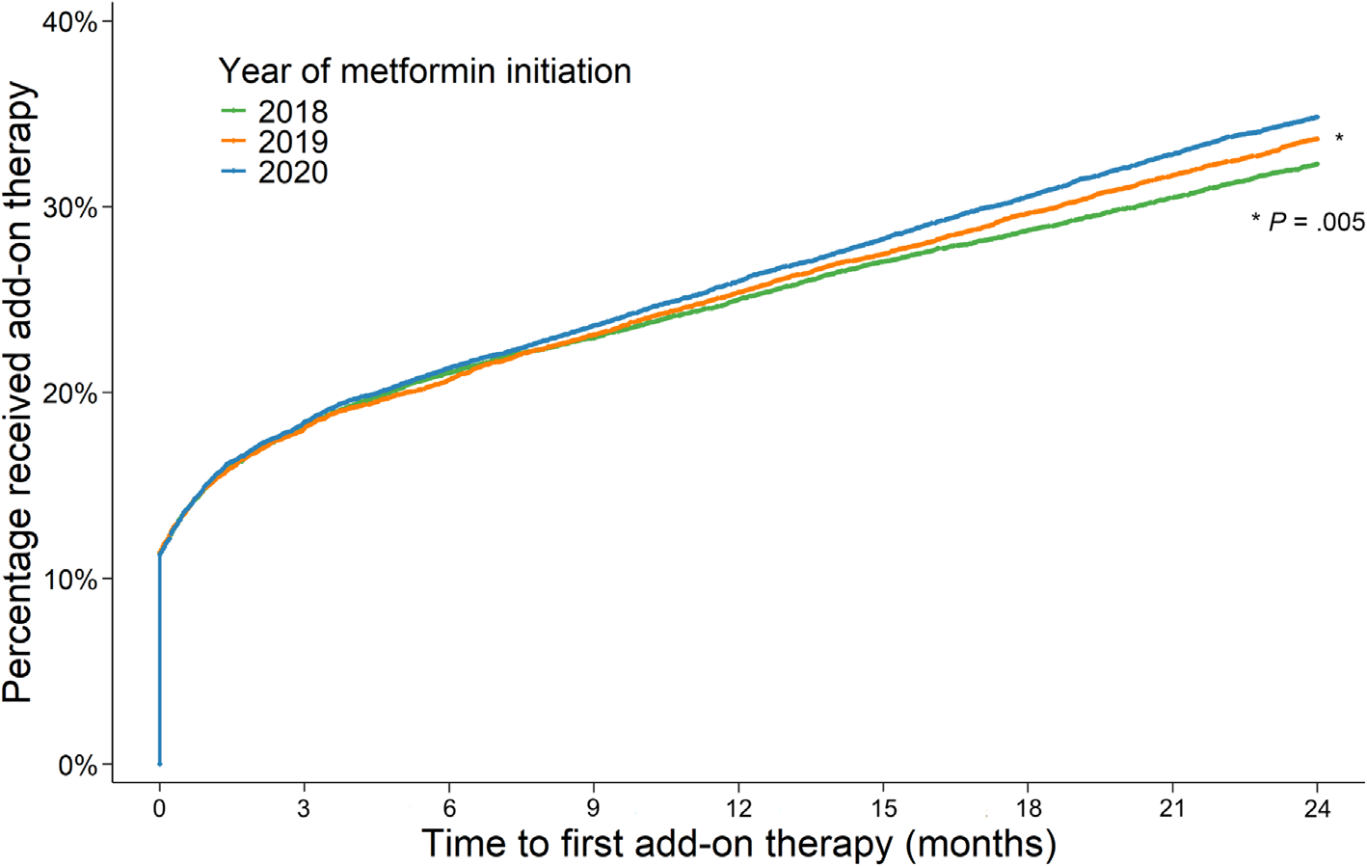
Proportion of study population who received add‐on therapy across the 2 years following metformin initiation, by year of metformin initiation.

Of people initiating metformin in 2018, 32.3% commenced add‐on anti‐hyperglycaemic therapy within 2 years (Figure 1). There was a statistically significant increase in the proportion of people initiating add‐on therapy in 2019 (33.7%) and 2020 (34.8%) (*P* = .005).

The characteristics of people who received add‐on therapy were different to those who did not (Table S2). People who received add‐on therapy tended to be younger (median age 59 years) and male (60%) compared to those who did not receive add‐on therapy (median age 61 years, 50% male).

Of people initiating metformin, the median time to add‐on therapy increased over time from 47 days in 2018, to 60 days in 2019 and 66 days in 2020. While the proportion of people receiving add‐on therapy in the first year of follow‐up was relatively consistent across years of metformin initiation, there was a significant increase in the proportion of people receiving add‐on therapy within the second year of initiation (*P* < .001) (Table S3).

Across all years, of people initiating add‐on therapy, approximately one‐third initiated combination add‐on therapy with metformin on the same day (2018: 35.3%; 2019: 34.0%; 2020: 32.6%). Excluding those initiated on combination therapy, of people initiating metformin in 2018, the median time to add‐on therapy was 212 days; 2019: 246 days; 2020: 251 days.

### 3.2 Type of first add‐on anti‐hyperglycaemic therapy

**FIGURE 2:**
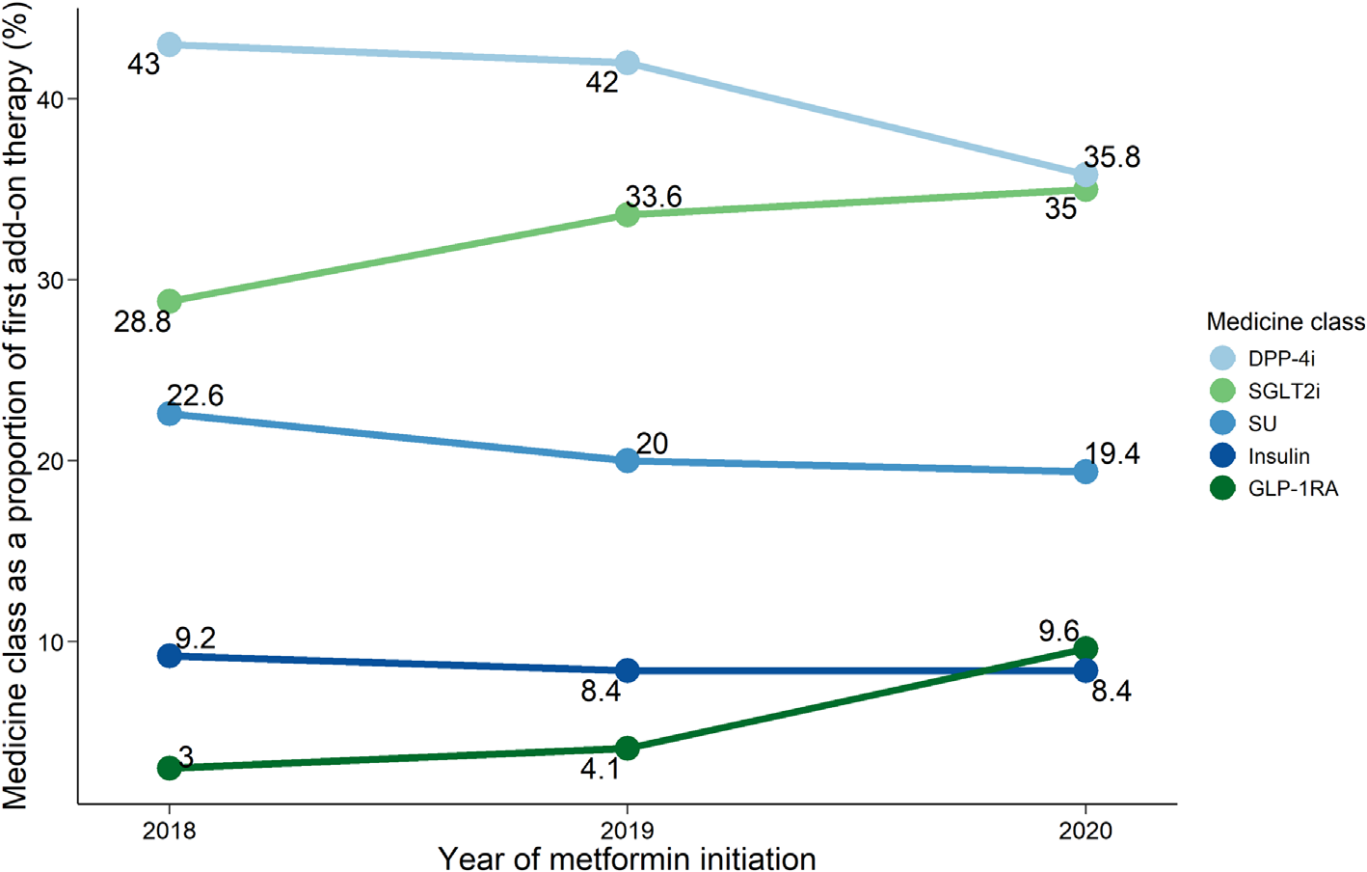
Type of first add‐on therapy by year of metformin initiation. Abbreviations: DPP‐4i, dipeptidyl peptidase‐4 inhibitors; SGLT2i, sodium‐glucose cotransporter 2 inhibitors; SU, sulfonylureas; GLP‐1 RA, glucagon‐like peptide‐1 receptor agonists.

We found the type of first add‐on anti‐hyperglycaemic therapy changed over the years (Figure 2). Amongst people initiating metformin in 2018, DPP‐4i were considerably more common as a first add‐on therapy (43.0%) compared to other medicines (28.8% for SGLT2i). Whereas amongst people initiating metformin in 2020, DPP‐4i and SGLT2i were used approximately equally (35.8% and 35.0% respectively). The proportion of GLP‐1 RA as first add‐on therapy tripled amongst people starting metformin in 2020 (9.6%) compared to 2018 (3.0%). Amongst people who initiated a combination of metformin and add‐on therapy on the same day, DPP‐4i remained the highest use first add‐on therapy across all 3 years of initiation (Table S4). Most of the initial combination therapy with DPP‐4i and SGLT2i and metformin was in the form of a multi‐medicine combination tablet rather than multiple single medicine tablets.

### 3.3 Differences by patient and prescriber characteristics

Use of first add‐on therapy in different age categories was largely consistent with the general trend (Table 2). However, in people aged 80 years and older, DPP‐4i remained the most common, representing approximately half of first add‐on therapy (Table 2). Second and third highest use medicine classes in this age category were sulfonylureas and SGLT2i, respectively, with low use of GLP‐1 RA.

**TABLE 2:** Types of first add‐on therapy, amongst people who received add‐on therapy within 2 years of metformin initiation, by age categories and year of metformin initiation.

Age categories (years) by year of metformin initiation	People with any add‐on therapy (*N*)	Type of add‐on therapy *n* (% of *N*)				
		SGLT2i	DPP‐4i	SU	GLP‐1 RA	Insulin
**40–49**						
2018	984	309 (31.4)	371 (37.7)	206 (20.9)	50 (5.1)	115 (11.7)
2019	979	343 (35.0)	389 (39.7)	182 (18.6)	54 (5.5)	97 (9.9)
2020	984	365 (37.1)	348 (35.4)	155 (15.8)	109 (11.1)	103 (10.5)
**50–59**						
2018	1287	392 (30.5)	564 (43.8)	267 (20.7)	35 (2.7)	108 (8.4)
2019	1303	488 (37.5)	525 (40.3)	244 (18.7)	67 (5.1)	106 (8.1)
2020	1417	517 (36.5)	485 (34.2)	217 (15.3)	176 (12.4)	107 (7.6)
**60–69**						
2018	1058	326 (30.8)	469 (44.3)	234 (22.1)	25 (2.4)	82 (7.8)
2019	1138	386 (33.9)	481 (42.3)	227 (19.9)	42 (3.7)	89 (7.8)
2020	1224	436 (35.6)	442 (36.1)	274 (22.4)	111 (9.1)	76 (6.2)
**70–79**						
2018	577	134 (23.2)	252 (43.7)	163 (28.2)	9 (1.6)	50 (8.7)
2019	590	169 (28.6)	273 (46.3)	130 (22.0)	7 (1.2)	42 (7.1)
2020	713	239 (33.5)	251 (35.2)	174 (24.4)	40 (5.6)	67 (9.4)
**80+**						
2018	214	25 (11.7)	117 (54.7)	62 (29.0)	<5	22 (10.3)
2019	212	33 (15.6)	105 (49.5)	63 (29.7)	<5	20 (9.4)
2020	266	56 (21.1)	121 (45.5)	73 (27.4)	5 (1.9)	33 (12.4)

*Note*: Data less than five are not shown due to data confidentiality. The sum of individual types of add‐on therapy may be greater than the number of people with add‐on therapy due to people using more than one type of first add‐on therapy.

Abbreviations: DPP‐4i, dipeptidyl peptidase‐4 inhibitors; GLP‐1 RA, glucagon‐like peptide‐1 receptor agonists; SGLT2i, sodium‐glucose cotransporter 2 inhibitors; SU, sulfonylureas.

There was an increase in the use of SGLT2i and GLP‐1 RA as first add‐on therapy over the years for both females and males (Table S5). However, SGLT2i use was greater amongst males compared with females, whereas GLP‐1 RA use was greater amongst females compared with males. Furthermore, amongst females initiating metformin in 2020, the highest use of first add‐on therapy was DPP‐4i, whereas it was SGLT2i for males.

Amongst people with evidence of treatment for cardiovascular disease (hypertension, hyperlipidaemia and/or use of antiplatelet medicines), the use of SGLT2i and GLP‐1 RA as first add‐on therapy was similar to the overall cohort (Table 3). There was a more pronounced change in people with congestive heart failure; in people starting metformin in 2020, SGLT2i use as a proportion of first add‐on therapy was 41.7% and DPP‐4i was 28.2%.

**TABLE 3:** Type of first add‐on therapy, amongst people who received add‐on therapy within 2 years of metformin initiation, by evidence of specific morbidities (derived through history of medicine dispensing) and year of metformin initiation.

Comorbidities by year of metformin initiation	People with any add‐on therapy (*N*)	Type of add‐on therapy, *n* (% of *N*)				
		SGLT2i	DPP‐4i	SU	GLP‐1 RA	Insulin
**Hypertension**						
2018	1224	336 (27.5)	529 (43.2)	294 (24.0)	33 (2.7)	116 (9.5)
2019	1283	450 (35.1)	507 (39.5)	239 (18.6)	54 (4.2)	112 (8.7)
2020	1426	497 (34.9)	501 (35.1)	305 (21.4)	137 (9.6)	110 (7.7)
**Hyperlipidaemia**						
2018	1933	576 (29.8)	832 (43.0)	467 (24.2)	44 (2.3)	160 (8.3)
2019	2038	717 (35.2)	872 (42.8)	392 (19.2)	77 (3.8)	155 (7.6)
2020	2301	873 (37.9)	804 (34.9)	466 (20.3)	211 (9.2)	159 (6.9)
**Congestive heart failure**						
2018	245	71 (29.0)	96 (39.2)	54 (22.0)	5 (2.0)	37 (15.1)
2019	294	112 (38.1)	102 (34.7)	52 (17.7)	13 (4.4)	38 (12.9)
2020	333	139 (41.7)	94 (28.2)	68 (20.4)	25 (7.5)	40 (12.0)
**Use of anti‐platelets**						
2018	274	79 (28.8)	108 (39.4)	80 (29.2)	9 (3.3)	35 (12.8)
2019	263	88 (33.5)	102 (38.8)	61 (23.2)	8 (3.0)	34 (12.9)
2020	330	120 (36.4)	94 (28.5)	77 (23.3)	27 (8.2)	43 (13.0)

*Note*: The sum of individual types of add‐on therapy may be greater than the number of people with add‐on therapy due to people using more than one type of first add‐on therapy.

Abbreviations: DPP‐4i, dipeptidyl peptidase‐4 inhibitors; GLP‐1 RA, glucagon‐like peptide‐1 receptor agonists; SGLT2i, sodium‐glucose cotransporter 2 inhibitors; SU, sulfonylureas.

The increase in SGLT2i and GLP‐1 RA as first add‐on therapy prescribed by primary care providers was similar to the overall study population (Table S6). SGLT2i and insulin were the first and second highest use first add‐on medicine classes prescribed by secondary care providers amongst people commencing metformin in 2020.

### 3.4 Sensitivity analysis

Changing the estimated period of exposure of metformin as the number of days in which 90% rather than 75% of people received a subsequent dispensing of metformin did not make a clinically meaningful difference to the proportion of the study population with add‐on therapy within 2 years of metformin initiation, or the median time to add‐on therapy (Table S7).

## 4 DISCUSSION

Amongst people initiating metformin between 2018 and 2020, approximately two‐thirds did not receive add‐on anti‐hyperglycaemic therapy within 2 years of metformin initiation. Of those who received add‐on therapy, there was an increase in the use of SGLT2i and GLP‐1 RA as first‐add on medicines, which is consistent with national and international type 2 diabetes guidelines. Amongst people with add‐on therapy following metformin initiation, SGLT2i use increased from 28.8% (2018) to 35.0% (2020), and GLP‐1 RA use increased from 3.0% to 9.6%, respectively. One‐third of add‐on therapy occurred on the same day metformin was initiated, i.e. initial combination therapy. Excluding those with initial combination therapy, the median time to add‐on therapy was approximately 8 months.

While our findings of increased uptake of SGLT2i and GLP‐1 RA are broadly consistent with international studies, the differences between countries highlight the potential role of medicines reimbursement policy on early use of these medicines.^24^, ^25^ The magnitude of change in SGLT2i use in our study most closely aligns with the United Kingdom compared with the United States.^15^ Out‐of‐pocket costs for SGLT2i and GLP‐1 RA in the United States can be high,^26^ potentially limiting use for some people within these settings. Furthermore, certain countries and provinces such as France and British Columbia in Canada have only subsidized SGLT2i in recent years, with evidence showing an uptake in use since their subsidization approval.^27^, ^28^

In addition to being able to prescribe subsidized medicines with cardiorenal benefits, clinicians also need to understand the rationale for use of these medicines and be confident to manage side effects.^24^, ^29^, ^30^ In a qualitative study of general practitioners, identified barriers to SGLT2i initiation included limited appreciation of the cardiorenal benefits of these medicines and the increasing complexity of type 2 diabetes pharmacotherapy.^31^ However, in this utilization study there was a clear trend of increased prescribing of SGLT2i and GLP‐1 RA by primary care providers, demonstrating some diffusion of updated guideline recommendations into primary care practice. Nevertheless, there was still high utilization of medicines without cardiorenal benefits—DPP‐4i and sulfonylureas, which highlights that it takes time for clinicians to adopt newer therapies as clinicians may be more comfortable with older medicines.^29^, ^31^ Clinicians may also prefer DPP‐4i and sulfonylureas over SGLT2i because of concerns about potential adverse effects and use of SGLT2i in people with more advanced CKD (despite the evidence for major cardiorenal benefits in this population).^29^, ^31^, ^32^

We found a high use of initial combination therapy, higher than that found in Danish, British Columbia and Catalonia cohorts.^28^, ^33^, ^34^ Our finding is particularly interesting given that the PBS restricts SGLT2i, DPP‐4i or GLP‐1 RA prescription to patients with a glycated haemoglobin (HbA1c) measurement of greater than 7% (53 mmol/mol) despite treatment with metformin or a sulfonylurea.^17^ There is a strong clinical rationale for using SGLT2i and GLP‐1 RA in the initial treatment of type 2 diabetes in high‐risk individuals because of the cardiorenal benefits of these medicines (reflected in guidelines from the American Diabetes Association and European Association for the Study of Diabetes since 2019).^1^, ^10^, ^35^, ^36^ Initial combination therapy with an SGLT2i or GLP‐1 RA overcomes therapeutic inertia and delay in the use of these medicines.^37^, ^38^ This is critically important as two‐thirds of our study population did not have add‐on therapy within 2 years of metformin initiation, which is consistent with the international study.^15^ Given the popularity of initial combination therapy with metformin in our study, this approach should undergo review by the government to determine whether removing the need for failure on metformin or a sulfonylurea to enable subsidization for an SGLT2i or GLP‐1 RA is cost‐effective. Similarly, removal of the requirement for an HbA1c of >7% (53 mmol/mol) may increase uptake in people with controlled diabetes, but at risk of cardiorenal complications.

Related to initial combination therapy, early adoption of SGLT2i and GLP‐1 RA in the disease course of type 2 diabetes is key to maximize the benefits of these agents.^11^, ^12^, ^39^ Hence, time to add‐on of SGLT2i or GLP‐1 RA in type 2 diabetes, explored in this study and not previous extensively studied, could be a valuable measure for comparison at a local level, for example hospital clinics, and at national and international levels.

Compared with other age categories, amongst those aged 80 years and over, there was lower use of SGLT2i and higher use of DPP‐4i and sulfonylureas, consistent with other studies.^15^, ^40^, ^41^, ^42^, ^43^ This may be related to safety concerns with SGLT2i in the elderly and warrants further study, given the lack of cardiovascular benefits with DPP‐4i and sulfonylureas and the risk of hypoglycaemia with sulfonylureas.^40^ Consistent with other literature was the preference for SGLT2i amongst men and GLP‐1 RA amongst women,^42^, ^44^, ^45^ the reasons for which need greater elucidation.

The major strength of our study is the use of a 10% nationwide dispensing sample ensuring representability of the entire country. PBS restrictions apply to all Australian states and territories and SGLT2i had been subsidized as first add‐on anti‐hyperglycaemic therapy during the entire study period. Hence, the trends observed largely reflect changing preferences of practitioners. There is a need for metformin therapy to enable PBS subsidization of SGLT2i and GLP‐1 RA, and whilst there would have been people who received SGLT2i or GLP‐1 RA without metformin on private scripts, the total number of such people should be low because of the lack of subsidization. Additionally, GLP‐1 RA are only listed on the PBS for type 2 diabetes, not for weight loss independent of diabetes and therefore changes observed should specifically relate to use in diabetes.

Our study had several limitations. The PBS dataset does not contain information on the indication for medicine use and we rely on medicines as a surrogate for diseases, so there may be misclassification of diseases. The dataset does not contain clinical or laboratory data such as weight, HbA1c and renal function. Therefore, it is not possible to determine the effect of these variables on the use of first add‐on therapy. As private prescriptions are not captured by this dataset, we may have underestimated the proportion of the study population with add‐on therapy. During early 2022, dapagliflozin and empagliflozin were listed on the PBS for chronic heart failure, and in late 2022, dapagliflozin was listed for proteinuric CKD.^17^, ^46^ Thus, whilst SGLT2i were subsidized for heart failure and CKD, because our study required that all individuals were using concomitant metformin, SGLT2i use was likely for the treatment of type 2 diabetes. The increase in SGLT2i use in people with evidence of comorbid congestive heart failure is consistent with type 2 diabetes guideline recommendations.^9^

Furthermore, we excluded adults younger than 40 years because many women are prescribed metformin for gestational diabetes and polycystic ovarian syndrome in this age group. We have therefore not captured young people with type 2 diabetes who are at very high risk of cardiovascular disease and premature mortality. We also note the occurrence of the COVID‐19 pandemic during the study period which had the potential to impact prescribing practice. Additionally, there was an international GLP‐1 RA shortage in 2022 which may have limited add‐on GLP‐1 RA dispensing that year.

In conclusion, there were rapid changes in the type of first add‐on anti‐hyperglycaemic therapy amongst people initiating metformin from 2018 to 2020 in Australia with increasing use of SGLT2i and GLP‐1 RA. However, only one‐third of the study population commenced add‐on therapy within 2 years of metformin initiation. Given the additional cardiorenal benefits of SGLT2i and GLP‐1 RA independent of glucose‐lowering, greater action is needed at the policy level to ease subsidized prescription of these medicines and to potentially increase prescribers' knowledge and confidence to initiate these medicines.

## AUTHOR CONTRIBUTIONS

T.Y.M., J.L., S.A.P., J.R.G., R.O.D., S.L.S., M.O.F. and J.O.C. were involved in the study conception and design. T.Y.M. conducted the data analysis under the supervision of J.L., M.O.F. and J.O.C. All authors were involved in the interpretation of the results. T.Y.M. drafted the manuscript, and all authors reviewed, edited and approved the final version of the manuscript.

## CONFLICT OF INTEREST STATEMENT

S.A.P. is a member of the Drug Utilization Sub‐Committee of the Pharmaceutical Benefits Advisory Committee. The views expressed in this paper do not represent those of the Committee. B.L.N. has received fees for travel support, advisory boards, scientific presentations and steering committee roles from AstraZeneca, Alexion, Bayer, Boehringer and Ingelheim, Cambridge Healthcare Research, Cornerstone Medical Education, Janssen, the limbic, and Medscape, with all honoraria paid to The George Institute for Global Health.

## Supporting information


**TABLE S1.** World Health Organization (WHO) Anatomic Therapeutic Chemical (ATC) codes for anti‐hyperglycaemic medicine classes of interest.
**Table S2.** Characteristics of people who received add‐on therapy compared to those who did not receive add‐on therapy, by year of metformin initiation.
**Table S3.** Proportion (%) of people having received add‐on therapy at different time points within 2 years of metformin initiation.
**Table S4.** Type of add‐on therapy when initiated with metformin on same day (initial combination therapy), by year of metformin initiation.
**Table S5.** Types of first add‐on therapy, amongst people who received add‐on therapy within 2 years of metformin initiation, by sex categories and year of metformin initiation.
**Table S6.** Type of first add‐on therapy amongst people who received add‐on therapy within 2 years of metformin initiation, by prescriber group and year of metformin initiation.
**Table S7.** Sensitivity analysis: proportion of people who received add‐on therapy within 2 years of metformin initiation, and median time to add‐on therapy, after changing the treatment exposure period of a metformin dispensing to the number of days in which 90% of people received a subsequent dispensing of metformin.
**Figure S1** Study design.
**Figure S2** Examples of identification of first add‐on therapy, using an estimated treatment exposure for each dispensing of metformin.

## Data Availability

The data were provided by the Australian Government. Access to the data by other individuals or authorities is not permitted without the express permission of the approving human research ethics committees and data custodians.
