# Supplementary material for: Use of, time to, and type of first add‐on anti‐hyperglycaemic therapy to metformin in Australia, 2018–2022
Source: Br J Clin Pharmacol. 2024 Sep 3;91(1):117–26. doi: 10.1111/bcp.16231 (PMC11671323; doi:10.1111/bcp.16231)
Supplement: Supplementary file 1 — TABLE S1. World Health Organization (WHO) Anatomic Therapeutic Chemical (ATC) codes for anti‐hyperglycaemic medicine classes of interest. Table S2. Characteristics of people who received add‐on therapy compared to those who did not receive add‐on therapy, by year of metformin initiation. Table S3. Proportion (%) of people having received add‐on therapy at different time points within 2 years of metformin initiation. Table S4. Type of add‐on therapy when initiated with metformin on same day (initial combination therapy), by year of metformin initiation. Table S5. Types of first add‐on therapy, amongst people who received add‐on therapy within 2 years of metformin initiation, by sex categories and year of metformin initiation. Table S6. Type of first add‐on therapy amongst people who received add‐on therapy within 2 years of metformin initiation, by prescriber group and year of metformin initiation. Table S7. Sensitivity analysis: proportion of people who received add‐on therapy within 2 years of metformin initiation, and median time to add‐on therapy, after changing the treatment exposure period of a metformin dispensing to the number of days in which 90% of people received a subsequent dispensing of metformin. Figure S1 Study design. Figure S2 Examples of identification of first add‐on therapy, using an estimated treatment exposure for each dispensing of metformin. [file BCP-91-117-s001.docx]

**Supplementary Table 1.** World Health Organisation (WHO) Anatomic Therapeutic Chemical (ATC) codes for anti-hyperglycaemic medicine classes of interest

| **Medicine class** | **WHO ATC code** | **Examples of medicine** |
| --- | --- | --- |
| Biguanides - metformin | A10BA02 | Metformin |
| Metformin combinations: |  |  |
| + Sulfonylureas | A10BD02 | Glibenclamide |
| + DPP-4i | A10BD07, A10BD08, A10BD10, A10BD11, A10BD13 | Sitagliptin, vildagliptin |
| + SGLT2i | A10BD15, A10BD20, A10BD23 | Dapagliflozin, empagliflozin |
| Sulfonylureas | A10BB | Glibenclamide, gliclazide, |
| DPP-4i | A10BH | Sitagliptin, vildagliptin |
| DPP-4i + SGLT2i | A10BD19, A10BD21, A10BD24 | Linagliptin + empagliflozin, saxagliptin + dapagliflozin |
| GLP-1 RA | A10BJ | Liraglutide, semaglutide |
| SGLT2i | A10BK | Dapagliflozin, empagliflozin, |
| Insulin | A10A | Insulin aspart, insulin glargine |

Abbreviations: DPP-4i, dipeptidyl peptidase-4 inhibitors; SGLT2i, sodium-glucose cotransporter 2 inhibitors; GLP-1 RA, glucagon-like peptide-1 receptor agonists

**Supplementary Table 2.** Characteristics of people who received add-on therapy compared to those who did not receive add-on therapy, by year of metformin initiation

| Characteristics by year of metformin initiation | People with add-on therapy | People with no add-on therapy |
| --- | --- | --- |
| **2018** | 4,120 | 8,707 |
| Age, years, median (IQR) | 58.2 (50.5-67.3) | 60.9 (51.6-69.9) |
| Age categories, years, n (%) |  |  |
| 40-49 | 984 (23.9) | 1,842 (21.2) |
| 50-59 | 1,287 (31.2) | 2,293 (26.3) |
| 60-69 | 1,058 (25.7) | 2,425 (27.9) |
| 70-79 | 577 (14.0) | 1,551 (17.8) |
| 80+ | 214 (5.2) | 596 (6.9) |
| Sex, n (%) |  |  |
| Female | 1,676 (40.7) | 4,366 (50.1) |
| Male | 2,444 (59.3) | 4,341 (49.9) |
| Concessional beneficiaries, n (%) | 1,912 (46.4) | 4,187 (48.1) |
| **2019** | 4,222 | 8,391 |
| Age, years, median (IQR) | 58.7 (50.6-67.5) | 60.9 (51.6-69.8) |
| Age categories, years, n (%) |  |  |
| 40-49 | 979 (23.2) | 1,803 (21.5) |
| 50-59 | 1,303 (30.9) | 2,155 (25.7) |
| 60-69 | 1,138 (27.0) | 2,368 (28.2) |
| 70-79 | 590 (14.0) | 1,491 (17.8) |
| 80+ | 212 (5.0) | 574 (6.8) |
| Sex, n (%) |  |  |
| Female | 1,661 (39.3) | 4,207 (50.1) |
| Male | 2,561 (60.7) | 4,184 (49.9) |
| Concessional beneficiaries, n (%) | 1,991 (47.2) | 3,951 (47.1) |
| **2020** | 4,604 | 8,703 |
| Age, years, median (IQR) | 59.2 (51.2-68.2) | 61.2 (51.8-70.4) |
| Age categories, years, n (%) |  |  |
| 40-49 | 984 (21.4) | 1,793 (20.6) |
| 50-59 | 1,417 (30.8) | 2,261 (26.0) |
| 60-69 | 1,224 (26.6) | 2,380 (27.3) |
| 70-79 | 713 (15.5) | 1,642 (18.9) |
| 80+ | 266 (5.8) | 627 (7.2) |
| Sex, n (%) |  |  |
| Female | 1,842 (40.0) | 4,355 (50.0) |
| Male | 2,762 (60.0) | 4,348 (50.0) |
| Concessional beneficiaries, n (%) | 2,226 (48.3) | 4,181 (48.0) |

**Supplementary Table 3.** Proportion (%) of people having received add-on therapy at different time points within two years of metformin initiation

| Days relative to metformin initiation | Year of metformin initiation | | | p-value (log-rank test) |
| --- | --- | --- | --- | --- |
|  | 2018 | 2019 | 2020 |  |
| Same day as metformin initiation | 11.3 | 11.4 | 11.3 | 0.969 |
| +90 days | 18.1 | 17.9 | 18.3 | 0.751 |
| +180 days | 21.0 | 20.6 | 21.3 | 0.440 |
| +365 days | 25.0 | 25.4 | 26.0 | 0.188 |
| +730 days | 32.3 | 33.7 | 34.8 | <0.001 |

**Supplementary Table 4.** Type of add-on therapy when initiated with metformin on same day (initial combination therapy), by year of metformin initiation

| Type of add-on therapy (number with initial combination therapy = n) | % of n |
| --- | --- |
| 2018 (n=1445) |  |
| SGLT2i | 322 (22.8) |
| DPP-4i | 730 (50.2) |
| SU | 384 (26.4) |
| GLP-1 RA | 24 (1.6) |
| Insulin | 161 (11.1) |
| 2019 (n=1437) |  |
| SGLT2i | 401 (27.9) |
| DPP-4i | 709 (49.3) |
| SU | 348 (24.2) |
| GLP-1 RA | 37 (2.6) |
| Insulin | 174 (12.1) |
| 2020 (n=1503) |  |
| SGLT2i | 458 (30.5) |
| DPP-4i | 708 (47.1) |
| SU | 358 (23.8) |
| GLP-1 RA | 29 (1.9) |
| Insulin | 210 (14.0) |

*Note*: The sum of individual types of add-on therapy may be greater than the number of people with add-on therapy due to people using more than one type of first add-on therapy.

Abbreviations: SGLT2i, sodium-glucose cotransporter 2 inhibitors; DPP-4i, dipeptidyl peptidase-4 inhibitors; SU, sulfonylureas; GLP-1 RA, glucagon-like peptide-1 receptor agonists

**Supplementary Table 5.** Types of first add-on therapy, amongst people who received add-on therapy within two years of metformin initiation, by sex categories and year of metformin initiation

| Sex categories by year of metformin initiation | People with any add-on therapy (n) | Type of add-on therapy (% of n) | | | | |
| --- | --- | --- | --- | --- | --- | --- |
|  |  | SGLT2i | DPP-4i | SU | GLP-1 RA | Insulin |
| **Female** |  |  |  |  |  |  |
| 2018 | 1676 | 443 (26.4) | 718 (42.8) | 376 (22.4) | 76 (4.5) | 158 (9.4) |
| 2019 | 1661 | 520 (31.3) | 676 (40.7) | 348 (21.0) | 103 (6.2) | 151 (9.1) |
| 2020 | 1842 | 553 (30.0) | 667 (36.2) | 358 (19.4) | 241 (13.1) | 154 (8.4) |
| **Male** |  |  |  |  |  |  |
| 2018 | 2444 | 743 (30.4) | 1055 (43.2) | 556 (22.7) | 46 (1.9) | 219 (9.0) |
| 2019 | 2561 | 899 (35.1) | 1097 (42.8) | 498 (19.4) | 69 (2.7) | 203 (7.9) |
| 2020 | 2762 | 1060 (38.4) | 980 (35.5) | 535 (19.4) | 200 (7.2) | 232 (8.4) |

*Note*: The sum of individual types of add-on therapy may be greater than the number of people with add-on therapy due to people using more than one type of first add-on therapy.

Abbreviations: SGLT2i, sodium-glucose cotransporter 2 inhibitors; DPP-4i, dipeptidyl peptidase-4 inhibitors; SU, sulfonylureas; GLP-1 RA, glucagon-like peptide-1 receptor agonists

**Supplementary table 6.** Type of first add-on therapy amongst people who received add-on therapy within two years of metformin initiation, by prescriber group and year of metformin initiation

*Note*: The sum of individual types of add-on therapy may be greater than the number of people with add-on therapy due to people using more than one type of first add-on therapy.

Abbreviations: SGLT2i, sodium-glucose cotransporter 2 inhibitors; DPP-4i, dipeptidyl peptidase-4 inhibitors; SU, sulfonylureas; GLP-1 RA, glucagon-like peptide-1 receptor agonists

| Prescriber group | People with any add-on therapy (n) | Type of add-on therapy (% of n) | | | | |
| --- | --- | --- | --- | --- | --- | --- |
|  |  | SGLT2i | DPP-4i | SU | GLP-1 RA | Insulin |
| **Primary care** |  |  |  |  |  |  |
| 2018 | 2388 | 691 (28.9) | 1095 (45.9) | 525 (22.0) | 63 (2.6) | 157 (6.6) |
| 2019 | 2453 | 818 (33.3) | 1093 (44.6) | 474 (19.3) | 104 (4.2) | 159 (6.5) |
| 2020 | 3031 | 1111 (36.7) | 1090 (36.0) | 555 (18.3) | 326 (10.8) | 160 (5.3) |
| **Secondary care** |  |  |  |  |  |  |
| 2018 | 341 | 104 (30.5) | 92 (27.0) | 71 (20.8) | 18 (5.3) | 101 (29.6) |
| 2019 | 312 | 84 (26.9) | 84 (26.9) | 67 (21.5) | 24 (7.7) | 95 (30.4) |
| 2020 | 354 | 113 (31.9) | 88 (24.9) | 63 (17.8) | 62 (17.5) | 91 (25.7) |
| **Unknown/undisclosed** |  |  |  |  |  |  |
| 2018 | 1391 | 391 (28.1) | 586 (42.1) | 336 (24.2) | 41 (2.9) | 119 (8.6) |
| 2019 | 1457 | 517 (35.5) | 596 (40.9) | 305 (20.9) | 44 (3.0) | 100 (6.9) |
| 2020 | 1219 | 389 (31.9) | 479 (39.3) | 275 (22.6) | 53 (4.3) | 135 (11.1) |

**Supplementary Table 7.** Sensitivity analysis**:** Proportion of people who received add-on therapy within two years of metformin initiation, and median time to add-on therapy, after changing the treatment exposure period of a metformin dispensing to the number of days in which 90% of people received a subsequent dispensing of metformin

| Year of metformin initiation | People who received add-on therapy, n (%) | Median time to add-on therapy (days), IQR |
| --- | --- | --- |
| 2018 | 4,156 (32.4) | 50 (0, 325) |
| 2019 | 4,249 (33.7) | 63 (0, 357) |
| 2020 | 4,642 (34.9) | 70 (0,365) |

Abbreviation: IQR interquartile range

**Supplementary Figure 1** Study design


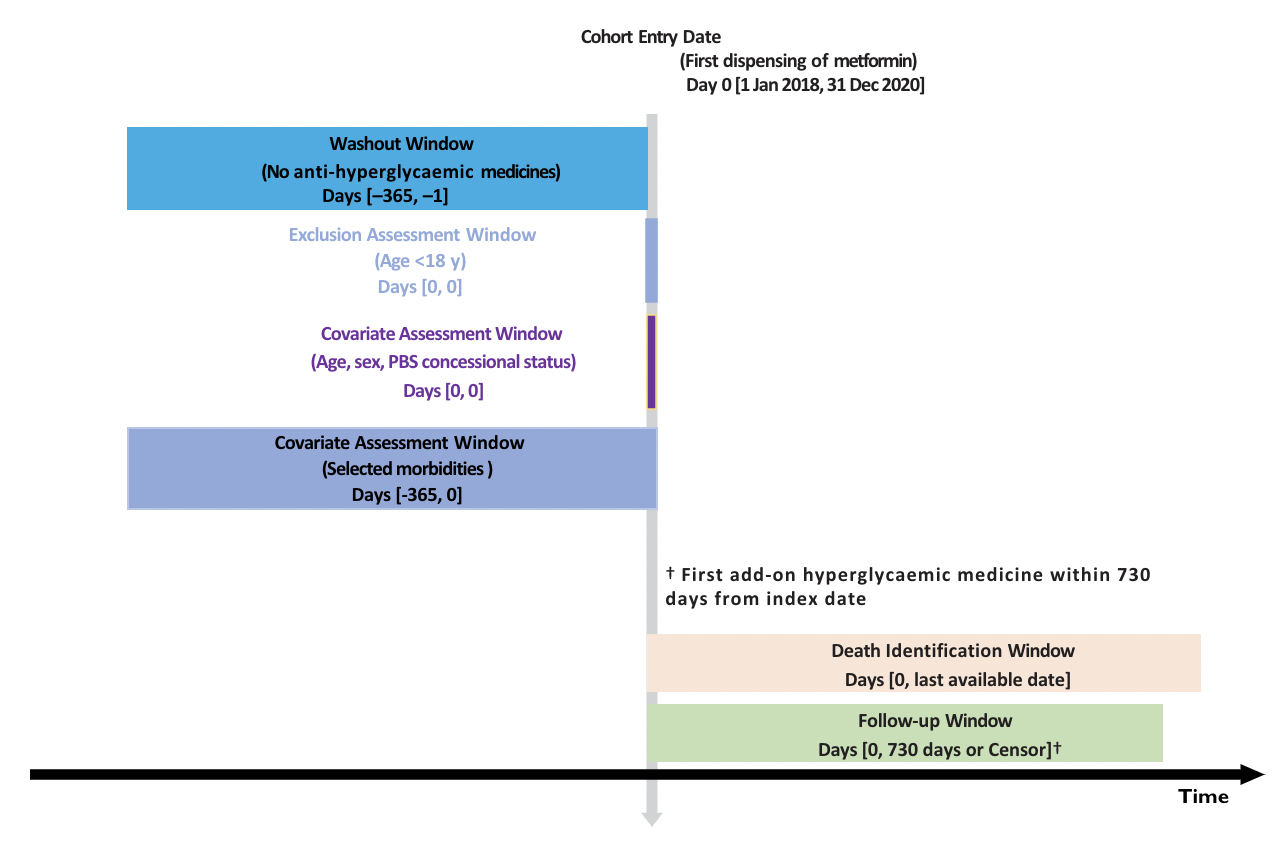


**Supplementary Figure 2** Examples of identification of first add-on therapy, using an estimated treatment exposure for each dispensing of metformin


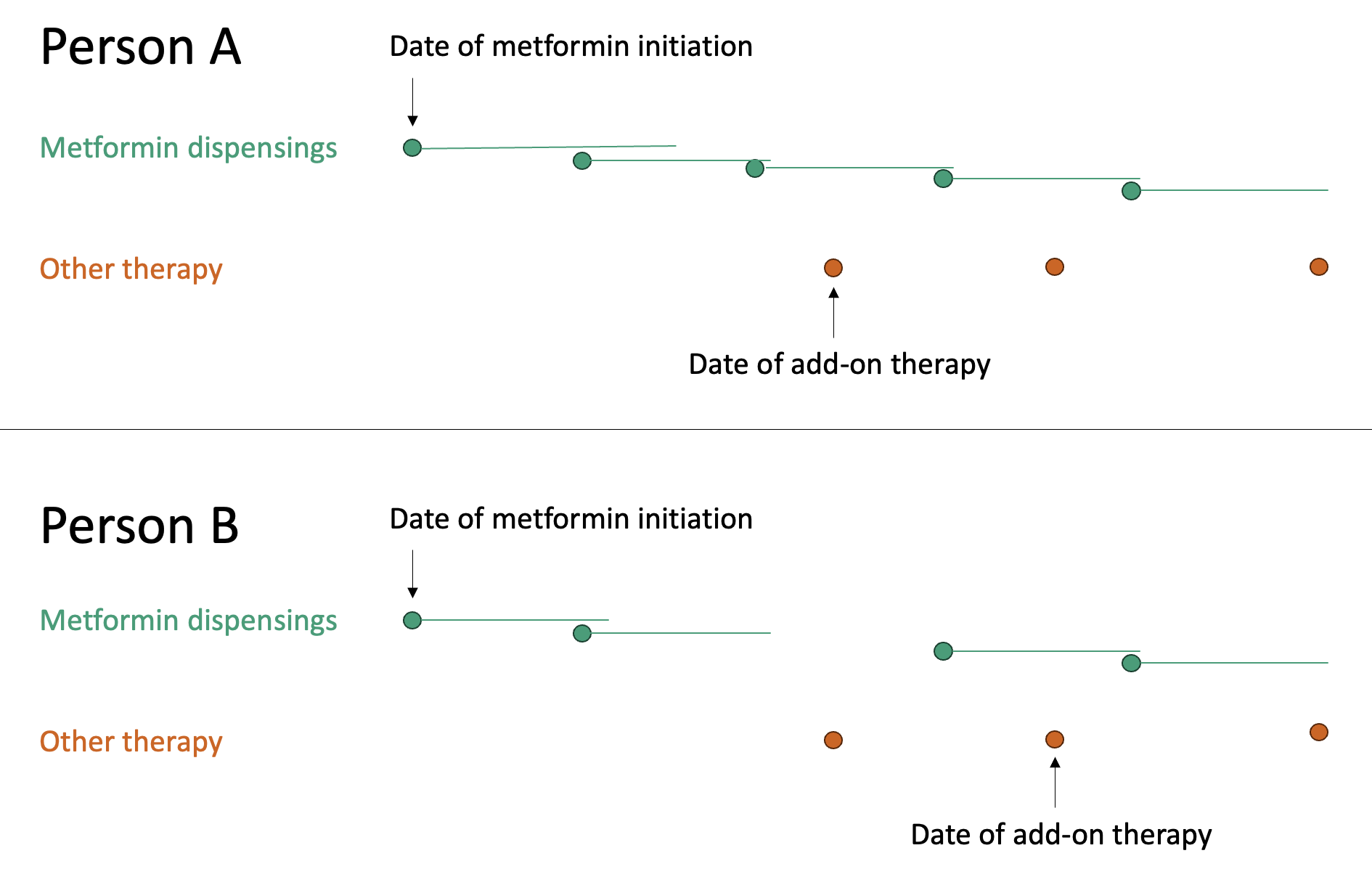


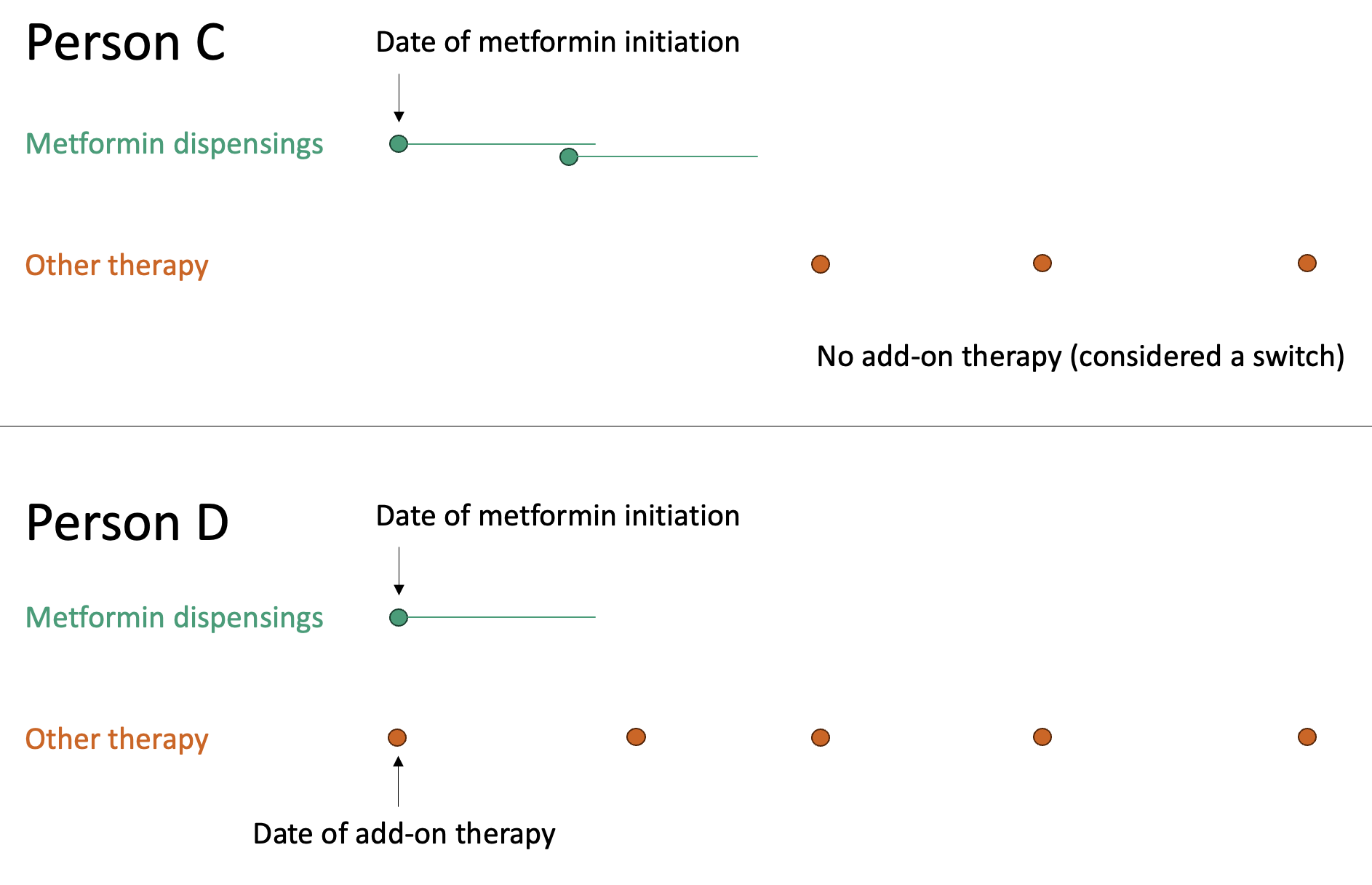


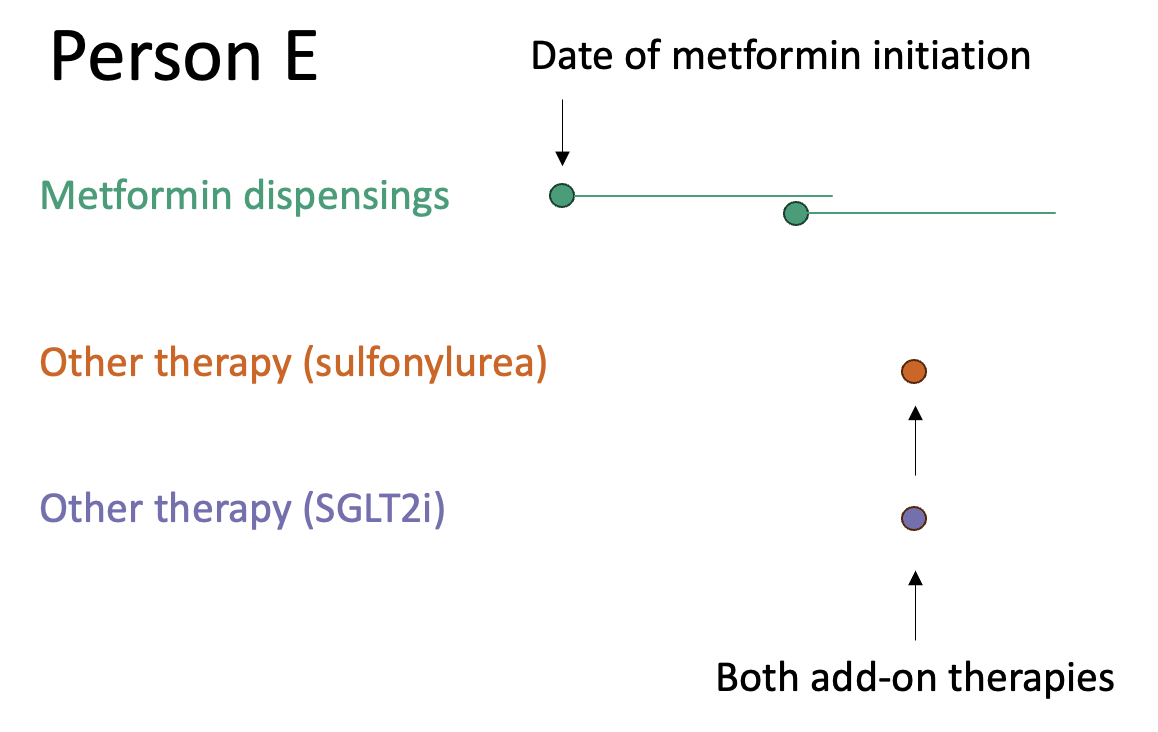


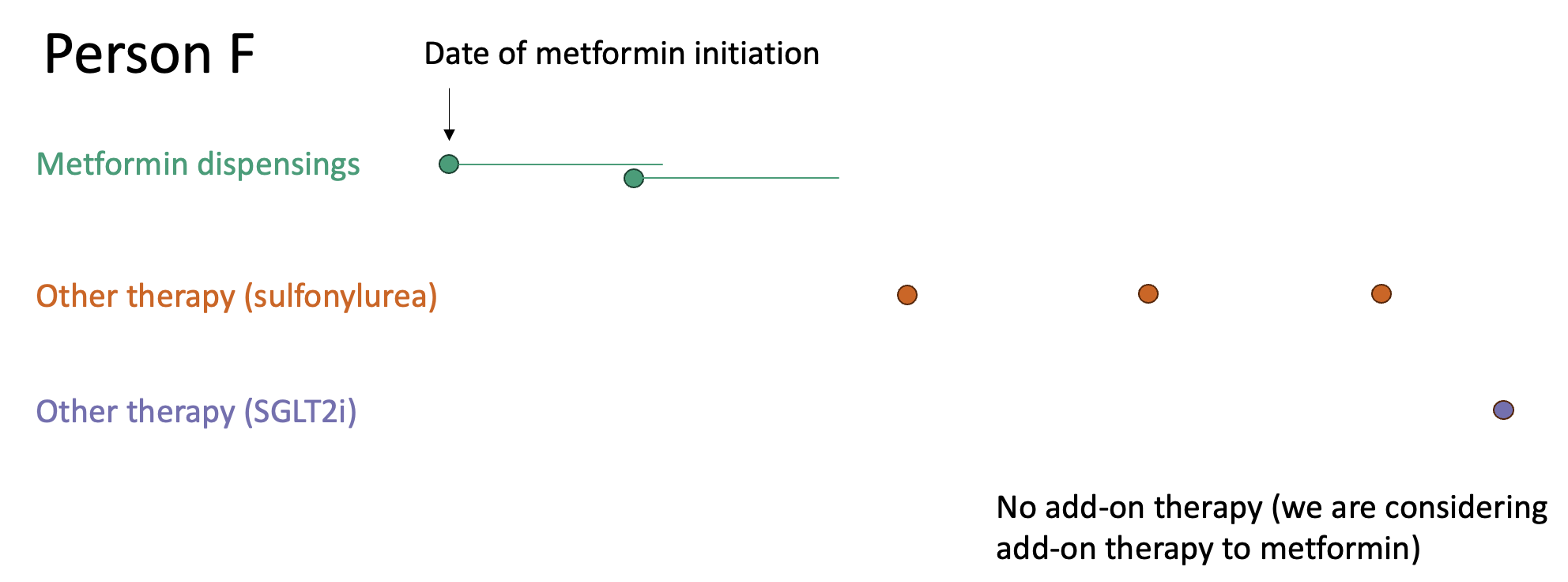


*Note*: The green line represents the duration of metformin exposure.
